# A new subspecies of Dactylolabis (Coenolabis) posthabita (Bergroth, 1888) from Hungary and new faunistic records of crane flies (Diptera, Tipuloidea) from the Balkans

**DOI:** 10.3897/zookeys.1291.199961

**Published:** 2026-09-14

**Authors:** Levente-Péter Kolcsár, Micha Camiel d’Oliveira, Wolfram Graf, Tibor Kovács, Dávid Murányi, Marija Ivković

**Affiliations:** 1 Gábor Áron 75, Ditrău, Romania Unaffiliated Ditrău Romania; 2 Nederlandse Entomologische Vereniging, Haarlem, Netherlands Nederlandse Entomologische Vereniging Haarlem Netherlands; 3 Institut für Hydrobiologie und Gewässermanagement, Wien, Austria Institut für Hydrobiologie und Gewässermanagement Wien Austria; 4 Mátra Museum of the Hungarian Natural History Museum of the Hungarian National Museum Public Collection Centre, Gyöngyös, Hungary Mátra Museum of the Hungarian Natural History Museum of the Hungarian National Museum Public Collection Centre Gyöngyös Hungary; 5 Eszterházy Károly University, Leányka u. 6, H-3300 Eger, Eger, Hungary Eszterházy Károly University Eger Hungary; 6 Samarkand State University named after Sharof Rashidov, Samarkand, Uzbekistan Samarkand State University named after Sharof Rashidov Samarkand Uzbekistan; 7 Department of Biology, Faculty of Science, University of Zagreb, Horvatovac 102A, 10000 Zagreb, Croatia Department of Biology, Faculty of Science, University of Zagreb Zagreb Croatia

**Keywords:** Balkans, faunistics, Hungary, Limoniidae, morphological variation, Tipulidae

## Abstract

The crane fly fauna (Diptera, Tipuloidea) of the Balkans remains poorly known, despite recent faunistic and taxonomic research. Here, we present new faunistic data on Limoniidae and Tipulidae from the region. In total, 20 species are reported from Albania, 15 from Montenegro, seven from North Macedonia, three from Bosnia and Herzegovina, three from Serbia, one from Croatia, and one from Bulgaria. Male genitalia of several rare or poorly known species are photographed for the first time. Morphological variation among populations of the rare and poorly known Dactylolabis (Coenolabis) posthabita (Bergroth, 1888) is discussed and illustrated, and a new subspecies, D. (C.) posthabita tothi Kolcsár, **ssp. nov**., is described from Hungary.

## Introduction

The crane fly fauna (Diptera, Tipuloidea) of the Balkans remains poorly known, despite the increasing number of faunistic and taxonomic studies published over recent decades. Kolcsár et al. (2021, 2023a) summarised faunistic and taxonomic advances, as well as new records published between 2010 and 2022 in Europe, including the Balkans, and also reported additional records from the region. Kolcsár et al. (2023b) described a new species and provided further faunistic records from the Balkans, while de Jong and Pavićević (2024) published new records of *Chionea* (Limoniidae) from the region. More recently, Jancsó et al. (2026) reported additional faunistic records of Tipuloidea from the Balkans.

In the present study, we provide additional faunistic data on Limoniidae and Tipulidae from the Balkans. In total, 20 species are recorded from Albania, 15 from Montenegro, seven from North Macedonia, three from Bosnia and Herzegovina, three from Serbia, and one each from Croatia and Bulgaria. Several rare or poorly known species have their male genitalia illustrated with photographs, many of which are published here for the first time. The observed morphological variation among populations of the rare and poorly known Dactylolabis (Coenolabis) posthabita (Bergroth, 1888) is discussed, and a new subspecies D. (C.) posthabita tothi Kolcsár, ssp. nov., is described. The female terminalia are described and illustrated for the first time, and a key to the species of the subgenus is provided.

## Materials and methods

The specimens were collected using entomological nets, aspirators, light traps, and pyramid emergence traps and were preserved in 70% ethanol or stored in triangular glassine envelopes. The vast majority of the specimens were identified by L.-P. Kolcsár, with some identifications by M. C. d’Oliveira. The genital structures were examined and photographed using a Zeiss Stemi 305 stereomicroscope and an AmScope B120 microscope equipped with a Canon 90D digital camera. Image stacks were combined using Zerene Stacker software. The terminology used in the descriptions of the male and female terminalia follows Kato (2023) and Ribeiro (2006).

Specimens from the following depositories were examined:

**CKLP** Private collection of L.-P. Kolcsár;

**HNHM** Hungarian National History Museum, Budapest, Hungary;

**PCMCO** Private Collection of M.C. d’Oliveira, Amsterdam, The Netherlands;

**MZH** Finnish Museum of Natural History Luomus, Helsinki, Finland.

Collectors:

**AA** – András Ambrus; **AE** – Anna Eichert; **AR** – A. Ries; **DM** – Dávid Murányi; **KF** – Kristóf Földi; **KH**– Krisztián Harmos; **LPK** – Levente-Péter Kolcsár; **MCO** – Micha Camiel d’Oliveira; **MI** – Marija Ivković; **PO** – Péter Olajos; **ET** – Edina Török; **TK** – Tibor Kovács; **WG** – Wolfram Graf.

## Results

### Taxonomic treatment

#### Subgenus Dactylolabis (Coenolabis)

Taxon classificationAnimaliaDipteraLimoniidae

Savchenko, 1969

B58F754A-48B7-5EAD-B730-01E714E20D05

##### Type species.

Dactylolabis (Coenolabis) aberrans Savchenko, 1963 by original designation in Savchenko (1969).

##### Remarks.

Prior to the present study, Dactylolabis (Coenolabis) comprised only four recognised species. *Dactylolabis arsianensis* Savchenko, 1969 and *D. imeretica* Savchenko, 1969 were originally described as subspecies of *D. aberrans* but are now treated as distinct species, due to their partial sympatry (Oosterbroek 2026). The subgenus is restricted to the Western Palearctic.

### Key to males of Dactylolabis (Coenolabis)

**Table:** 

1	Hypandrium medially membranous, appearing bifurcate, with tubercles bearing tufts of long setae (Savchenko 1969: fig. 2/2). Lateral projections of proctiger broaden apically (Savchenko 1969: fig. 2/1, interpreted and labeled as interbase)	**D. (C.) aberrans**
–	Hypandrium with a rounded median margin or a single median projection. Lateral projections of proctiger gradually tapering apically (Fig. 2)	**2**
2	Clasper of gonostylus long, arising from the middle of fused gonostyli (Savchenko 1969: fig. 3)	**D. (C.) arsianensis**
–	Clasper of gonostylus short, arising near apex of fused gonostyli	**3**
3	Interbase plate (including ventral lobes of interbase and medially fused portion) only slightly wider than aedeagus approx. 1.2× (Savchenko 1969: fig. 4)	**D. (C.) imeretica**
–	Interbase plate approx. 1.5–1.7× wider than aedeagus (Fig. 3D, I)	**4**
4	Median fused portion of interbase situated at approx. two-thirds the length of the aedeagal complex (Fig. 3D)	**D. (C.) posthabita posthabita**
–	Median fused portion of interbase situated at approx. half-length of the aedeagal complex (Fig. 3I)	**D. (C.) posthabita tothi Kolcsár, ssp. nov**.

#### Dactylolabis (Coenolabis) posthabita

Taxon classificationAnimaliaDipteraLimoniidae

(Bergroth, 1888)

4CF7A1ED-2C86-5C87-8772-9A5891CF21AE

##### Comments.

Dactylolabis (Coenolabis) posthabita is a very rare and poorly known species, originally described by Bergroth (1888) from Slovenia (Ljubljana) as *Limnophila posthabita*. Savchenko (1982) examined the type series (three males), redescribed the male genitalia, and transferred the species to *Dactylolabis*, tentatively placing it in the subgenus *Coenolabis*. However, this placement remained uncertain because the subgenus is primarily defined by female characters, while only males were known at that time.

Erhan-Dincă (1984) later reported the species from Romania (Eastern Carpathians), providing records of 16 males and 10 females and schematically illustrating the male terminalia. She referred to the species as *Coenolabis* without question and without further discussion. Savchenko (1986) later noted that he had received specimens from Erhan-Dincă, including females, and briefly mentioned that the ovipositor of the Romanian D. (C.) posthabita females is similar to that of D. (C.) aberrans, characterised by short hypogynial valves, although this was not illustrated. The species was later reported from Hungary by Kolcsár et al. (2021) based on five males. In addition, the first author located further material in the HNHM, including female specimens from Hungary. Erhan-Dincă (1984) reported 14 males and 10 females deposited in the Grigore Antipa National Museum of Natural History (Bucharest, Romania). However, the Romanian material now appears to be either lost or remains uncatalogued in the Diptera collection of the Grigore Antipa Museum (Melania Stan pers. comm.).

Comparison of three males and one female from Serbia with the male genitalia illustrations provided by Savchenko (1982, repeated in 1986), as well as those of Erhan-Dincă (1984), and with photographs of Hungarian specimens published by Kolcsár et al. (2021), together with additional Hungarian material, revealed several minor differences. The most notable difference is observed in the Hungarian and Serbian females, which possesses well-developed, elongate hypogynial valves reaching approx. half-length of the cerci, in contrast to the condition mentioned by Savchenko (1986) for the Romanian material. Unfortunately, due to the unavailability of Romanian specimens, direct comparison with the original material was not possible and the illustration provided by Erhan-Dincă (1984) is schematic and cannot be used for detailed comparison in case of males.

We compared the type material deposited in the MZH with specimens from Hungary and Serbia and found clear but relatively minor differences among the three populations, most notably in the Hungarian specimens (see discussion below). However, the examined material shows no distinct differences in habitus, coloration, wing venation, or antennal segmentation, a degree of morphological uniformity also observed in other species of *Coenolabis* (Savchenko 1969). Therefore, we treat the Hungarian population as a new subspecies rather than a distinct species, pending future genetic analyses that may clarify its taxonomic status and degree of differentiation.

#### Dactylolabis (Coenolabis) posthabita posthabita

Taxon classificationAnimaliaDipteraLimoniidae

(Bergroth, 1888)

059DDC90-6890-5E6A-B9F0-A4479C30C5C2

**Figure 1.**
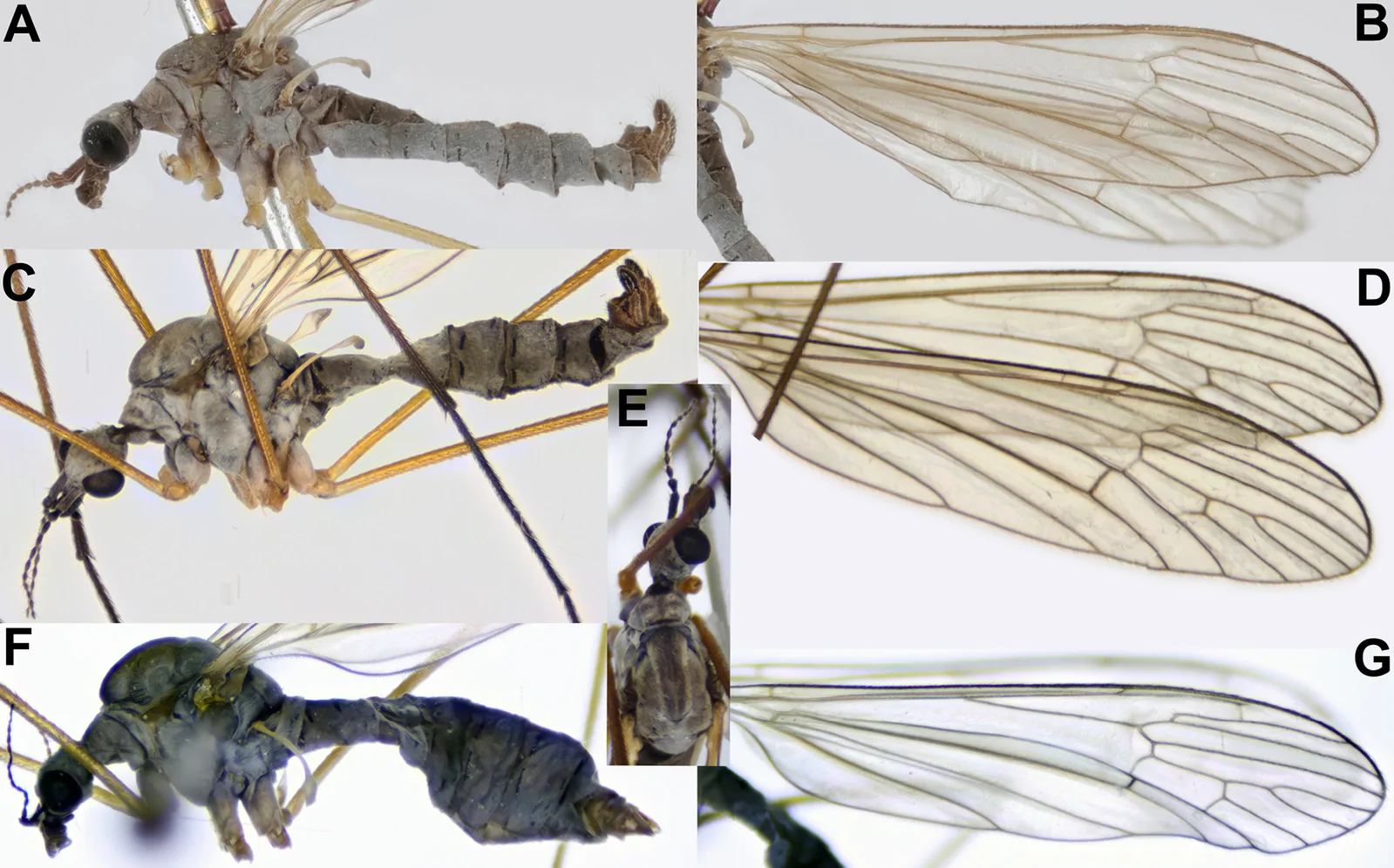
Dactylolabis (Coenolabis) posthabita (Bergroth, 1888) habitus. **A–E**. *D. p. posthabita*: **A, B**. Holotype male: Slovenia, Ljubljana, MZH: GV.28617; **C–E**. Male: Serbia, Kraljevo, CKLP: T154/1/3. **F, G**. *D. p. tothi* Kolcsár, ssp. nov.: paratype female: Hungary, Potony, HNHM. **A**, **C, F**. Habitus, lateral view; **B, D, G**. Wings; **E**. Head and thorax, dorsal view.

**Figure 2.**
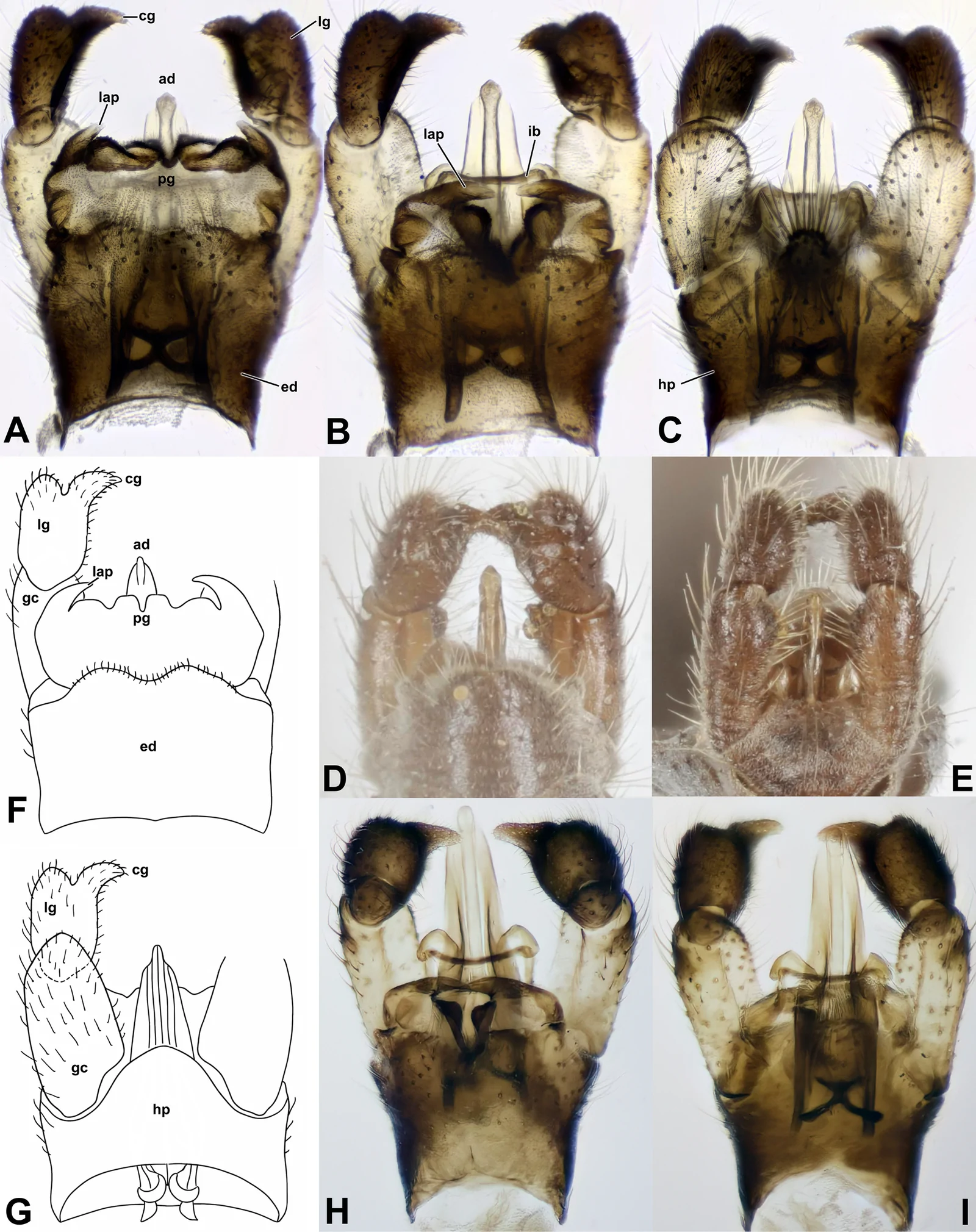
Dactylolabis (Coenolabis) posthabita (Bergroth, 1888) terminalia. **A–G**. *D. p. posthabita*: **A–C**. Male: Serbia, Kraljevo; CKLP: T154/1/2. **D, E**. Holotype male: Slovenia, Ljubljana, MZH: GV.28617; **F, G**. Schematic illustration after Savchenko (1986); **H, I**. *D. p. tothi* Kolcsár, ssp. nov.: paratype male: Hungary, Potony, HNHM; **A, B, D, F, H**. Male terminalia, dorsal view; **C, E, G, I**. Male terminalia, ventral view. Abbreviations: ad, aedeagus; cg, clasper of gonostylus; ed, epandrium; gc, gonocoxite; hp, hypandrium; ib, interbase; lap, lateral arm of proctiger; lg, lobe of gonostylus; pg, proctiger.

**Figure 3.**
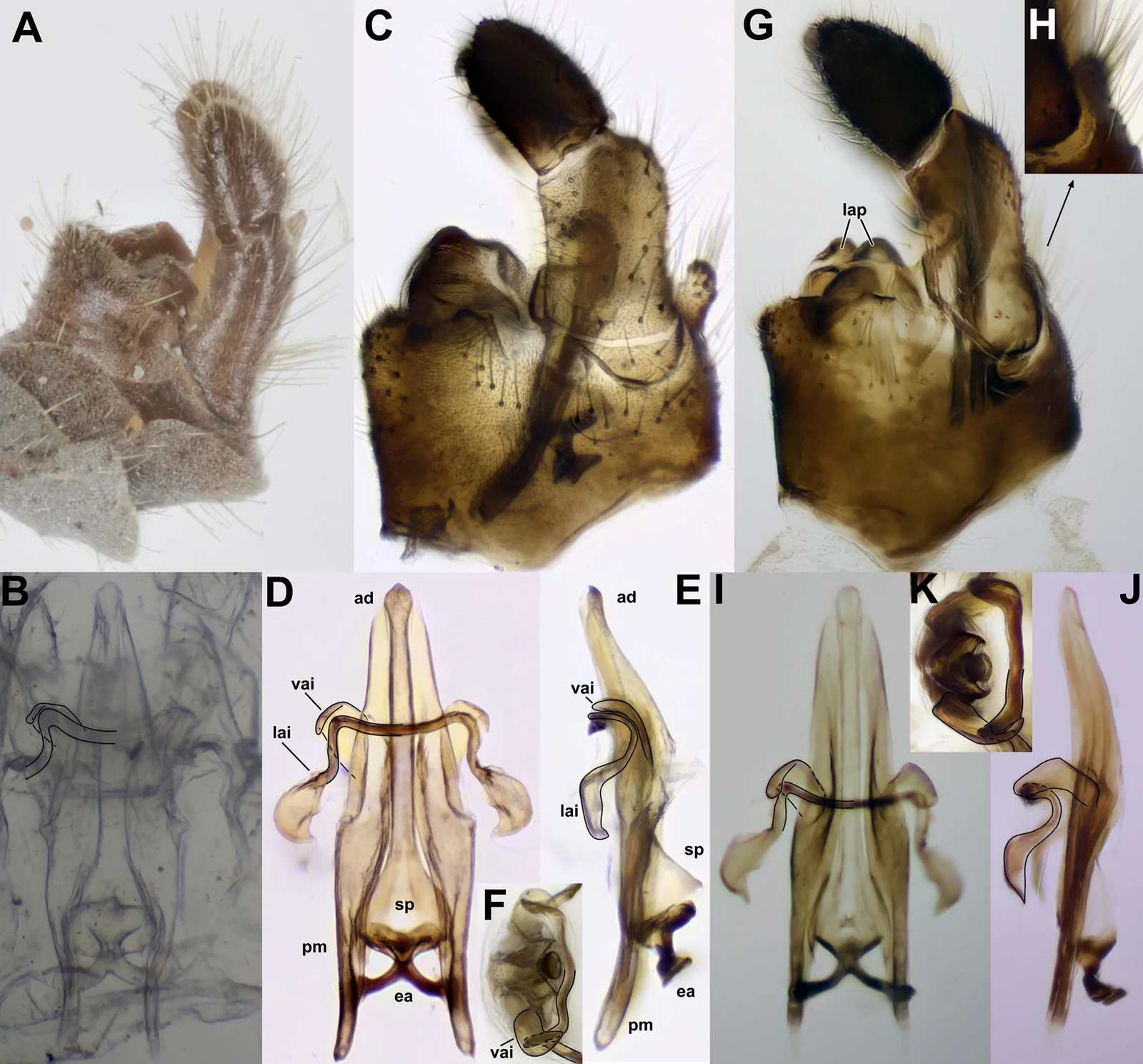
Dactylolabis (Coenolabis) posthabita (Bergroth, 1888) terminalia. **A–F**. *D. p. posthabita*: **A**. Holotype; **B**. Paratype: Slovenia, Ljubljana, MZH: GV.28617, GV.28618; **C–F**. Male: Serbia, Kraljevo, CKLP: T154/1/2; **G–K**. *D. p. tothi* Kolcsár, ssp. nov.: paratype: Hungary, Potony, HNHM; **A, C, G, H**. Male terminalia, lateral view; **B, D, I**. Aedeagal complex, dorsal view; **E, J**. Aedeagal complex, lateral view; **F, K**. Aedeagal complex, caudal view. Abbreviations: ad, aedeagus; ea, ejaculatory apodeme; lai, lateral arm of interbase; pm, paramere; sp, sperm pump; vai, ventral lobe of interbase.

**Figure 4.**
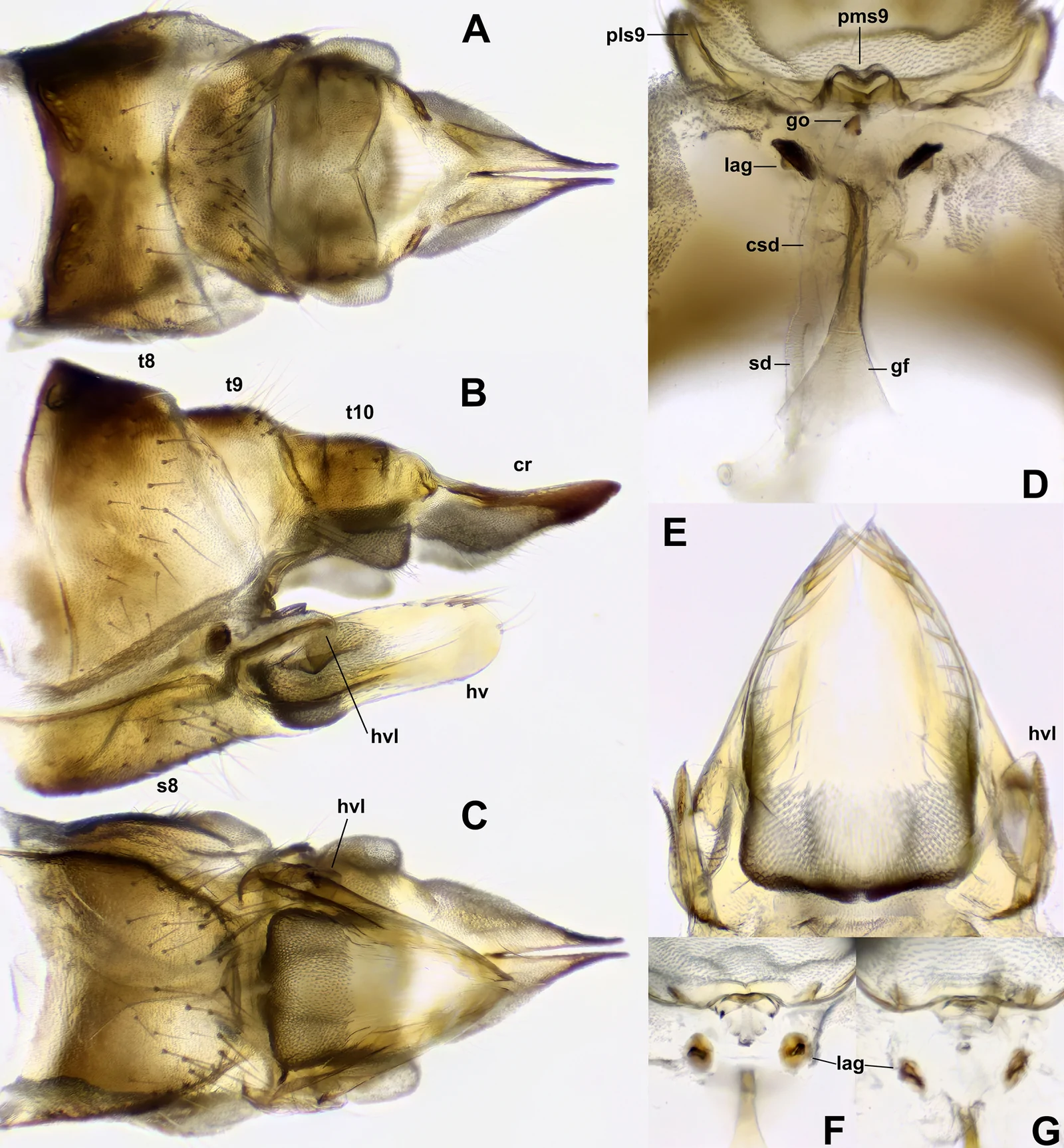
Dactylolabis (Coenolabis) posthabita (Bergroth, 1888) female. **A–F**. *D. p. posthabita* Serbia, Kraljevo, CKLP: T154/1/1; **G**. *D. p. tothi* Kolcsár, ssp. nov.; paratype Hungary, Pécs, Bányatelep, HNHM; **A**. Female terminalia, dorsal view; **B**. Female terminalia, lateral view; **C**. Female terminalia, ventral view; **D**. Sternite 9 and genital furca, ventral view; **E**. Sternite 8 and hypogynial valve, dorsal view; **F, G**. Sternite 9, caudal view. Abbreviations: cr, cercus; csd, common spermathecal duct; gf, genital fork; go, genital opening; hv, hypogynial valve; hvl, lobe of hypogynial valve; lag, lateral arm of genital frame; pls9, posterolateral lobe of sternite 9; pms9, posteromedial lobe of sternite 9; sd, spermathecal duct; s8, sternite 8; t8–t10, tergites 8–10.

Figs 1A–E, 2A–G, 3A–F, 4A–F

##### Type material examined.

Slovenia • ***Holotype*** ♂; Laibach (Ljubljana); 1870; leg. Palmén, Johan Axel; MZH • ***Paratypes*** 2 ♂♂; same data as for holotype; MZH; http://id.luomus.fi/GV.28617.

##### Additional material.

Serbia • 3 ♂♂, 1 ♀; Raška district, Kraljevo city, Samaila, open oak forest and cemetery in a church garden; 43.7620°N, 20.5447°E; alt. 235 m a.s.l.; 7 May 2023; leg. LPK, TK, DM; CKLP.

##### Diagnosis.

Fused median part of interbase situated at two-thirds length of aedeagal complex (at half-length in *p. tothi* Kolcsár, ssp. nov.). Ventral arm of interbase drop-shaped, with a triangular apex in caudal view (rectangular, with a narrow, pointed apex in *p. tothi* Kolcsár, ssp. nov.); narrow, band-like, with an evenly curved posterior margin in lateral view (as wide as tip of aedeagus and bent almost perpendicularly at mid-length in *p. tothi* Kolcsár, ssp. nov.).

##### Redescription.

A detailed general description of the species was provided by Savchenko (1982, 1986) and is not repeated here; only the characters of the male and female terminalia are discussed below. The general appearance of the female is similar to that of the male.

***Male terminalia***. Epandrium fused with hypandrium, approx. as wide as long; posterior margin slightly convex, with small lateral lobe (Fig. 2A, B, H). Posterior margin of hypandrium variable in shape: in type material rounded and bearing several setae (Fig. 2E, G), whereas Serbian specimens possess a distinct, finger-like lobe with long setae (Figs 2C, 3C). In well-dissected specimens, the proctiger is fully inflated and roughly quadrate, with lateral lobes triangular, with obtuse apices directed approx. 45° inward (Fig. 2A, F). If median part of proctiger is slightly compressed, the lateral lobes may appear more perpendicularly directed inward (as in Fig. 2B). Gonocoxite (Fig. 2A–G) 1.7–1.9× longer than wide, slightly longer than epandrium. Gonostyli broadly fused (Fig. 2); lobe of gonostylus (outer gonostylus) balloon-shaped, clasper of gonostylus (inner gonostylus) flamingo-beak-shaped, arising near apex of fused gonostyli. Interbase (Fig. 3B, D) with median portion fused into a narrow band, situated at approx. 2/3 of length of aedeagal complex; lateral arm widened near gonocoxal apodeme, sinuate in lateral view (Fig. 3E). Ventral arm partially covering lateral arm in caudal view (Fig. 3F), short, drop-shaped, with ventral part directed inward at nearly a right angle; dorsal apex triangular. Ventral arm in lateral view (Fig. 3E) narrow, band-like, with posterior margin evenly curved. Paramere (Fig. 3B, D, E) slender, anterior end weakly curved outward. Aedeagus robust, dorsoventrally flattened, with well-visible median tube (Fig. 3B, D), slightly curved dorsally (Fig. 3E). Sperm pump drop-shaped, approx. half the length of aedeagus (Fig. 3B, D) and wider than aedeagus in lateral view (Fig. 3E). Ejaculatory apodeme (Fig. 3B, D, E) with arms widened outward and directed ventrolaterally.

***Female terminalia***. Tergite 10 roughly rectangular in dorsal view (Fig. 4A). Cercus (Fig. 4B) longer than tergite 10, basally widened, distally narrowing, curving upward in lateral view; apex blunt, without teeth or saw-tooth-like structures (Fig. 4B). Sternite 8 (Fig. 4C) slightly longer than wide. Hypogynial valves well developed, approx. as long as sternite 8; apex reaching approx. to mid-length of cercus (Fig. 4B). A small additional lobe near base of lateral margin of hypogynial valve, triangular in ventral view (Fig. 4C). Sternite 9 with anterior margin deeply notched medially; posterior margin with a central anteriorly directed lobe, posterolateral parts evenly curved, directed posteriorly (Fig. 4D). Genital fork widening anteriorly, sinuate in lateral view, as long as sternite 8 (Fig. 4C). Genital opening weakly sclerotised; common spermathecal duct as long as genital fork, bifurcating into two branches; no sclerotised spermathecae visible. Lateral arms of genital frame with central part distinctly produced, sombrero-shaped in ventral view (Fig. 4D), roughly circular in caudal view (Fig. 4F).

##### Comments.

Previously reported from Slovenia, the Hungarian record reported by Kolcsár et al. (2021) is transferred to *D. posthabita tothi* Kolcsár, ssp. nov., whereas the Romanian records remain tentative (see discussion below). First record from Serbia.

#### Dactylolabis (Coenolabis) posthabita tothi

Taxon classificationAnimaliaDipteraLimoniidae

Kolcsár ssp. nov.

48D35C06-03CF-5409-B374-F7C5D733FB4B

https://zoobank.org/106633DB-0C28-49EF-942C-3D85143332F1

Figs 1F, 1G, 2H, 2I, 3G–K

Dactylolabis (Coenolabis) posthabita (Bergroth, 1888) in Kolcsár et al. 2021.

##### Type material.

***Holotype*: Hungary** • ♂ (pinned); Potony; 45.93°N, 17.64°E (approx.; not stated on the label); 03 May 1977; leg. S. Tóth; HOLOTYPE Dactylolabis (Coenolabis) posthabita tothi Kolcsár, ssp. nov. [red label]; HNHM.

***Paratypes*: Hungary** • 6 ♂♂, 1 ♀ (pinned); same locality and date as holotype; HNHM • 3 ♂♂ (pinned); Darány, Barcsi-Ősborókás; 45.98°N, 17.563°E (approx.; not stated on the label); 03 May 1977; leg. S. Tóth; HNHM • 2 ♀♀ (pinned); Pécs, Bányatelep; 46.13°N, 18.33°E (approx.; not stated on the label); 05–07 June 1957; leg. F. Mihály; det. as *Dactylolabis tergestina* Egger, 1863 by Mannheims 1965; HNHM • 1 ♀ (pinned); Pécs, Bányatelep, gesztenyés felett; 46.13°N, 18.33°E (approx.; not stated on the label); 05–07 June 1957; leg. F. Mihály; det. as *Dactylolabis tergestina* Egger, 1863 by Mannheims 1965; HNHM • 1 ♂ (pinned); Pécs, Mecsek-hg., Jakob hg., tölgyes; 46.09°N, 18.14°E (approx.; not stated on the label); 05–07 June 1957; leg. F. Mihály; det. as *Dactylolabis tergestina* Egger, 1863 by Mannheims 1965; HNHM.

##### Diagnosis.

Fused median part of interbase situated at approx. half-length of aedeagal complex (at two-thirds length in *p. posthabita*). Ventral arm of interbase rectangular, with a narrow, pointed apex in caudal view (drop-shaped, with a triangular apex in *p. posthabita*); as wide as tip of aedeagus and bent almost perpendicularly at mid-length in lateral view (narrow, band-like, with an evenly curved posterior margin in *p. posthabita*).

##### Description.

General appearance and size as nominate subspecies in both sexes (Fig. 1).

***Male terminalia***. In general (Fig. 2H, I, 3G, H), similar to the nominate subspecies, differing as follows: interbase (Fig. 3I) with fused median part situated at approx. half-length of aedeagal complex; in caudal view (Fig. 3K) ventral arm of interbase longer than in nominate subspecies, nearly rectangular, directed more ventrally at approx. 45° with dorsal apex narrow, pointed; in lateral view (Fig. 3J), as wide as apex of aedeagus, posterior margin bent almost perpendicularly at mid-length. Sperm pump as wide as aedeagus in lateral view (Fig. 3J).

**Female**. General appearance as male.

***Female terminalia***. In general, similar to the nominate subspecies, differing as follows: lateral arms of genital frame elongate, approx. twice as long as wide in caudal view (Fig. 4G).

##### Etymology.

The subspecies name is dedicated to the memory of the recently deceased Sándor Tóth (1930–2024), a Hungarian entomologist, who collected the majority of the types. The name is a noun in genitive case and masculine form.

##### Discussion.

The male genitalia of *D. posthabita* are very similar to those of *D. imeretica*, suggesting a closer morphological affinity between these taxa than between *D. imeretica* and *D. aberrans*. However, according to Savchenko (1969), females of *D. imeretica* cannot be distinguished from those of *D. aberrans* and therefore possess short hypogynial valves. In contrast, females of both subspecies of *D. posthabita* have distinctly elongate hypogynial valves. The two subspecies mainly differ in the shape and position of the ventral arm of the interbase. While the interbase appears identical in the paratype of *p. posthabita* from Slovenia and the specimen from Serbia, the posterior margin of the hypandrium differs; in the type material it is rounded in dorsal view (Fig. 2E, G), whereas in the Serbian specimens it is produced into a finger-like lobe, clearly visible in lateral view (Fig. 3C), which is also characteristic of *D. imeretica*. The Hungarian *p. tothi* Kolcsár, ssp. nov., shows an intermediate condition, with the posterior margin slightly produced (Fig. 3H). Other observed differences visible in the figures, such as the shape of the proctiger, appear to result largely from deformation or slight differences in orientation. The wing venation of the examined specimens of both subspecies shows variation in the position of m–cu relative to the fork of M (Fig. 1B, D, G). However, this character is highly variable among specimens and may even vary between the left and right wings of the same individual, suggesting that it is not associated with subspecific or population-level differences.

The Hungarian specimens were collected in Potony, most likely along the Drava River or in a relatively natural forest patch in 1977 (Sándor Tóth pers. comm. 2018), with additional specimens collected a few kilometres further in an old natural *Juniperus*-dominated, open, sandy steppe nature reserve. Further specimens were collected in the Mecsek Mountains in oak and sweet chestnut forests. The Serbian specimens were collected near a small graveyard with large oak trees in the surrounding area; one male was observed resting on mosses at the base of a concrete fence. Both the Hungarian *p. tothi* Kolcsár, ssp. nov., and Serbian *p. posthabita* specimens were collected from early May to early June. The exact collection date of the Slovenian type material of *p. posthabita* is unknown; only the year is available.

Erhan-Dincă (1984) reported that the Romanian specimens were collected in a wet, well-forested montane habitat between mid-September and late October. If the hypogynial valves of these specimens are indeed short, as stated by Savchenko (1986), then, considering both their occurrence at higher elevations and their markedly different flight period (autumn rather than late spring to early summer, as observed in other populations), the Romanian population could represent a distinct species and warrants further investigation.

### Faunistic records

The general distribution of the species is not discussed here; only their occurrence in the Balkans (**AL**: Albania; **BiH**: Bosnia and Herzegovina; **BG**: Bulgaria; **HR**: Croatia; **GR**: mainland of Greece; **XK**: Kosovo; **ME**: Montenegro; **MK**: North Macedonia; **RS**: Serbia; and **TR**: the European part of Turkey) is mentioned and are based on Oosterbroek (2026).

### Limoniidae

**1. Antocha (Orimargula) alpigena (Mik, 1883)**

**Material examined. Montenegro** • 1 ♂; Mojkovac municipality, Sinjavina Mts, spring and outlet along road P4; 42.99265°N, 19.48441°E; alt. 910 m a.s.l.; 21 June 2023; leg. EKCU field practice; CKLP.

**Comments**. Previously reported from BiH and BG. First record from ME.

**2. Cheilotrichia (Empeda) cinerascens (Meigen, 1804)**

**Material examined. Albania** • 1 ♂; Petran, Lëngarica Canyon; 40.249876°N, 20.444836°E; alt. 350 m a.s.l.; 12 October 2023; leg. LPK; CKLP. • 1 ♂; Tepelenë, Bënçë River; 40.2878°N, 20.0081°E; alt. 240 m a.s.l.; 10 October 2023; leg. LPK; CKLP. **Bosnia and Herzegovina** • 1 ♂, 1 ♀; Republika Srpska, Sutjeska, forest spring outlet along the Tjentište–Ćurevo road; 43.35866°N, 18.70809°E; alt. 820 m a.s.l.; 4 May 2023; leg. LPK, TK, DM; CKLP.

**Comments**. Previously reported from BG and RS. First record from AL and BiH.

**3. Cheilotrichia (Empeda) neglecta (Lackschewitz, 1927)**

**Material examined**. Montenegro • 1 ♂; Milogora, small temporary pond; 43.2174°N, 18.9285°E; alt. 1465 m a.s.l.; 5 October 2023; leg. LPK; CKLP.

**Comments**. First record from ME and from the Balkans.

**4. Crypteria (Crypteria) limnophiloides Bergroth, 1913**

**Material examined. Albania** • 6 ♂♂, 4 ♀♀; Frashër, Fir of Hotova National Park, springs; 40.344998°N, 20.395324°E; alt. 1100 m a.s.l.; 11 October 2023; leg. LPK; CKLP.

**North Macedonia** • 1 ♀; Tresonche, Tresonce Waterfall; 41.5670°N, 20.7551°E; alt. 1340 m a.s.l.; 14 October 2023; leg. LPK; CKLP.

**Comments**. Previously reported from BG, ME, RS, and the TR. First record from AL and MK.

**5. Dicranophragma (Brachylimnophila) adjunctum (Walker, 1848)**

**Material examined. Albania** • 1 ♀; Pagri, Kamenckës Canyon; 40.3278°N, 20.3655°E; alt. 950 m a.s.l.; 11 October 2023; leg. LPK; CKLP • 1 ♀; Bogovë, Bogova Waterfall; 40.5679°N, 20.1688°E; alt. 335 m a.s.l.; 13 October 2023; leg. LPK; CKLP.

**Comments**. Previously reported from the TR. First records from AL.

**6. Ellipteroides (Photogonomyia) limbatus (Roser, 1840)**

**Material examined. Croatia** • 1 ♀; Ličko-Senjska, Plitvički Ljeskovac, Potok Napojiste, Plitvice NP; 44.826667°N, 15.616111°E; alt. 681 m a.s.l.; 15 July 2025; leg. MI; CKLP.

**Comments**. Previously reported from BiH, BG, ME, MK, and RS. First record from HR.

**7. Erioptera (Erioptera) fusculenta Edwards, 1938**

**Material examined. Albania** • 1 ♂; Tepelenë; 40.291°N, 20.026°E; alt. 130 m a.s.l.; 18 October 2024; leg. WG; CKLP • 1 ♂; Tepelenë; 40.291°N, 20.026°E; alt. 130 m a.s.l.; 24 September 2023; leg. WG; CKLP • 1 ♂; Tepelenë, Bënçë River; 40.2878°N, 20.0081°E; alt. 240 m a.s.l.; 10 October 2023; leg. LPK; CKLP. • 1 ♂; Bogovë, Bogova Waterfall; 40.5679°N, 20.1688°E; alt. 335 m a.s.l.; 13 October 2023; leg. LPK; CKLP.

**Comments**. Previously reported from BG, ME, MK, RS, and TR. First records from AL.

**8. Erioptera (Erioptera) griseipennis Meigen, 1838**

**Material examined. Montenegro** • 2 ♂♂; Šavnik municipality, Sinjajevina Mts, Tušina, Kikov Spring and its outlet; 42.94683°N, 19.1902°E; alt. 990 m a.s.l.; 4 May 2023; leg. LPK, TK, DM; CKLP.

**Comments**. Previously reported from BiH, BG, and RS. First record from ME.

**9. Erioptera (Erioptera) lutea Meigen, 1804**

**Material examined. Montenegro** • 5 ♂♂; Šavnik municipality, Sinjajevina Mts, Tušina, Kikov Spring and its outlet; 42.94683°N, 19.1902°E; alt. 990 m a.s.l.; 4 May 2023; leg. LPK, TK, DM; CKLP • 1 ♂; Gusinje municipality, Gusinje, Alipaši Springs; 42.5492°N, 19.8247°E; alt. 940 m a.s.l.; 6 May 2023; leg. LPK, TK, DM; CKLP.

**Comments**. Previously reported from BiH, BG, HR, GR, RS, and TR. First records from ME.

**10. Gonomyia (Gonomyia) conoviensis Barnes, 1924**

Fig. 5

**Figure 5.**
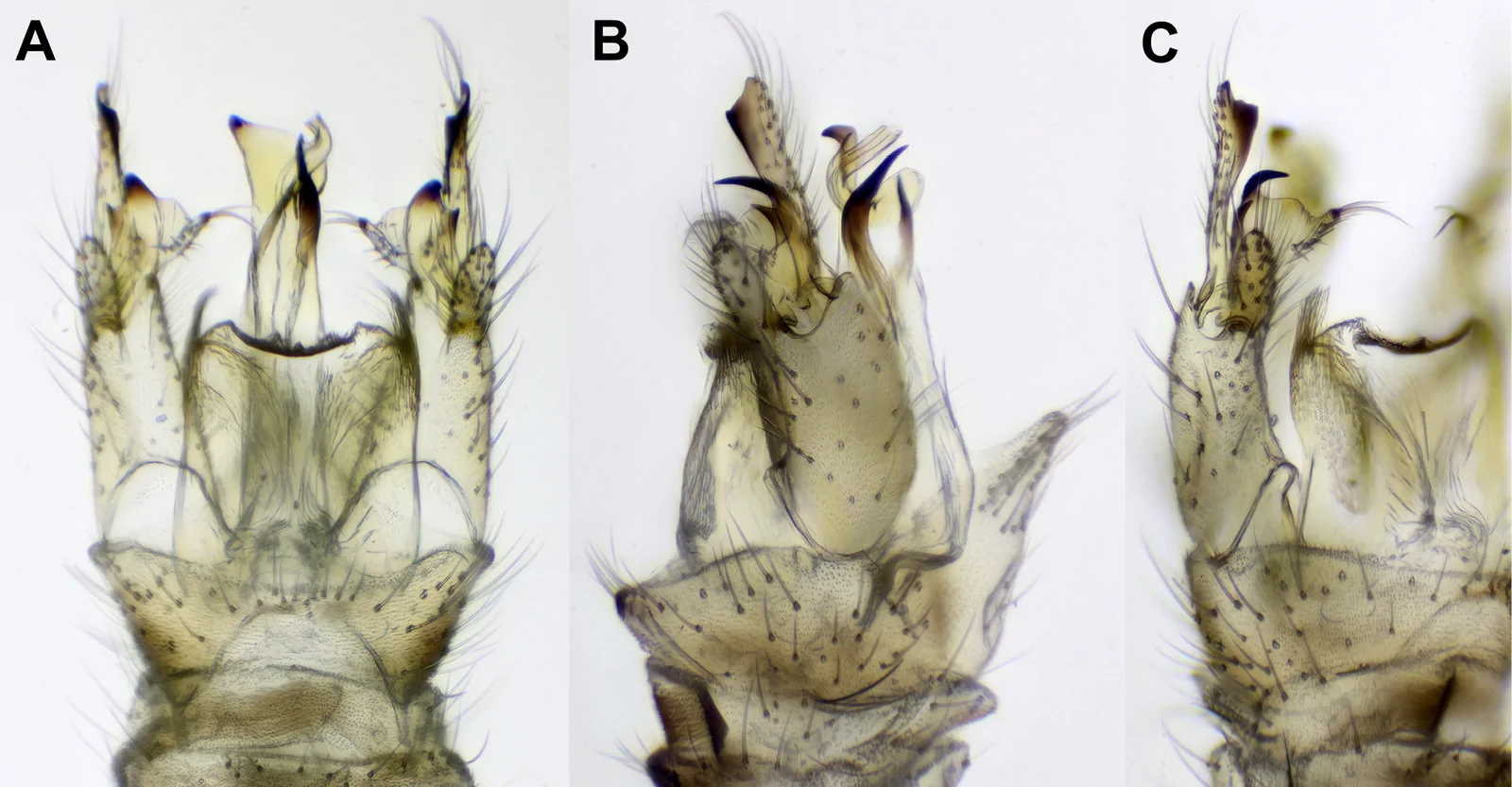
Gonomyia (Gonomyia) conoviensis Barnes, 1924 male: Albania, Selenicë, CKLP: T182/3/1. **A**. Male terminalia, dorsal view; **B**. Male terminalia, lateral view; **C**. Male terminalia, dorso-lateral view.

**Material examined. Albania** • 1 ♂; Selenicë, upper reach of Kokojkës River; 40.3341°N, 20.4388°E; alt. 1010 m a.s.l.; 11 October 2023; leg. LPK; CKLP.

**Comments**. Previously reported from BG, GR, ME, and TR. First record from AL.

**11. Gonomyia (Gonomyia) simplex Tonnoir, 1920**

**Material examined. Montenegro** • 1 ♂; Gusinje municipality, Gusinje, Alipaši Springs; 42.54918°N, 19.82473°E; alt. 940 m a.s.l.; 6 May 2023; leg. LPK, TK, DM; CKLP.

**Comments**. Previously reported from BG. First record from ME.

**12. Helius (Helius) pallirostris Edwards, 1921**

**Material examined. Albania** • 1 ♂; Poçem, Vjosa River; 40.50672°N, 19.72575°E; alt. 40 m a.s.l.; 14 June 2014; leg. WG; CKLP.

**Comments**. Previously reported from HR and TR. First record from AL.

**13. Hoplolabis (Eurasicesa) idiophallus (Savchenko, 1973)**

Fig. 6

**Figure 6.**
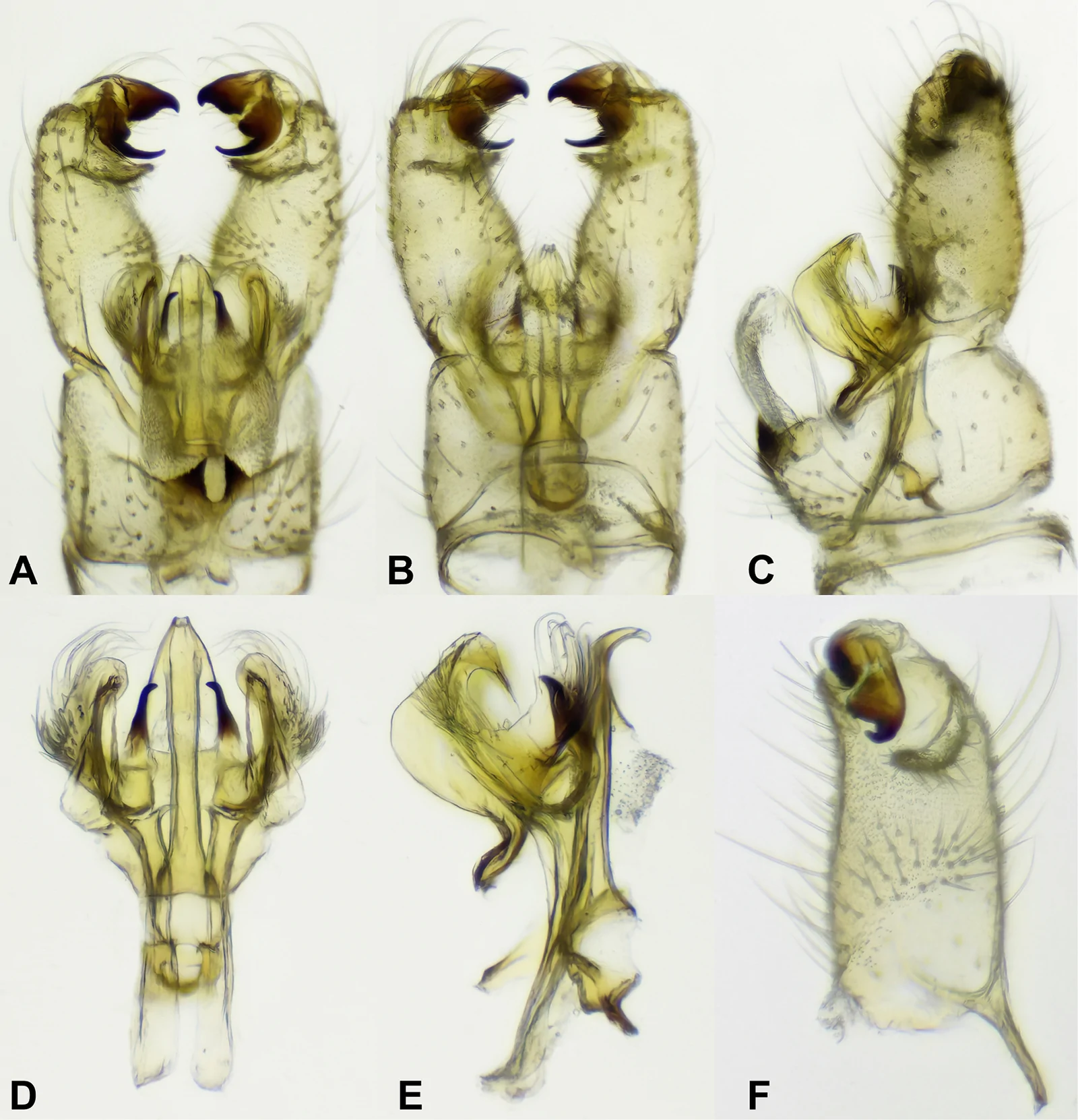
Hoplolabis (Eurasicesa) idiophallus (Savchenko, 1973) male: Albania, Tepelenë, CKLP: EULIM_10_035. **A–C**. Male terminalia; **A**. Dorsal view; **B**. Ventral view; **C**. Lateral view; **D, E**. Aedeagus complex; **D**. Dorsal view; **E**. Lateral view; **F**. Gonocoxite and gonostylus, inner lateral view.

**Material examined. Albania** • 25 ♂♂; Tepelenë, confluence of Vjosa and Drinos; 40.28034°N, 20.04429°E; alt. 130 m a.s.l.; 14 June 2014; leg. WG; CKLP • 9 ♂♂; Poçem, Vjosa River; 40.50672°N, 19.72575°E; alt. 40 m a.s.l.; 14 June 2014; leg. WG; CKLP • 2 ♂♂; Vjosa, Poçem; 40.509639°N, 19.731194°E; alt. 40 m a.s.l.; 13 May 2024; leg. WG; CKLP.

**Comments**. Previously reported from BG. First records from AL.

**14. *Limonia macrostigma* (Schummel, 1829)**

**Material examined. Montenegro** • 2 ♂♂, 2 ♀♀; Gusinje municipality, Gusinje, Alipaši Springs; 42.54918°N, 19.82473°E; alt. 940 m a.s.l.; 6 May 2023; leg. LPK, TK, DM; CKLP.

**Comments**. Previously reported from BG, HR, GR, XK, MK, RS, and TR. First record from ME.

**15. *Limonia nubeculosa* Meigen, 1804**

**Material examined. Albania** • 2 ♂♂, 3 ♀♀; Ilias, Gjipe Canyon; 40.148281°N, 19.678127°E; alt. 307 m a.s.l.; 29 June 2017; leg. LPK, ET; CKLP. • 3 ♂♂, 1 ♀; Ilias, Gjipe Canyon, in canyon without flowing water; 40.132°N, 19.675°E; 17 May 2024; MCO; PCMCO.

**Comments**. Previously reported from BiH, BG, HR, GR, ME, MK, RS and TR. First records from AL.

**16. *Lipsothrix ecucullata* Edwards, 1938**

**Material examined. North Macedonia** • 1 ♂; Southwestern, Plakenska Planina, Brežani, forest brook S of the village; 41.252389°N, 20.967389°E; alt. 1135 m a.s.l.; 27 June 2024; leg. TK, AA, KH; CKLP.

**Comments**. Previously reported from ME. First records from MK.

**17. *Lipsothrix remota* (Walker, 1848)**

**Material examined. Montenegro** • 1 ♂; Plav municipality, Visitor Mts, fir forest along Katun road; 42.6294°N, 19.8442°E; alt. 980–1515 m a.s.l.; 25 June 2023; leg. EKCU field practice; CKLP.

**Comments**. Previously reported from AL, BiH, BG, HR, GR, and MK. First records from ME.

**18. Molophilus (Molophilus) clavistylus Mendl, 1979**

**Material examined. Albania** • 1 ♂; Pagri, Kamenckës Canyon; 40.3278°N, 20.3655°E; alt. 950 m a.s.l.; 11 October 2023; leg. LPK; CKLP.

**Comments**. Previously reported from GR. First record from AL.

**19. Molophilus (Molophilus) czizeki Lackschewitz, 1931**

**Material examined. Bosnia and Herzegovina** • 5 ♂♂, 1 ♀; Republika Srpska, Sutjeska, forest spring outlet along the Tjentište–Ćurevo road; 43.35866°N, 18.70809°E; alt. 820 m a.s.l.; 4 May 2023; leg. LPK, TK, DM; CKLP.

**Serbia** • 1 ♂; Raška district, Novi Pazar municipality, Golija Mts, Tunovo, forest stream and sidebrook; 43.28262°N, 20.44081°E; alt. 850 m a.s.l.; 7 May 2023; leg. LPK, TK, DM; CKLP.

**Comments**. Previously reported from BG and ME. First record from BiH and RS.

**20. Molophilus (Molophilus) tjederi Starý, 1968**

**Material examined. Albania** • 1 ♂; Vjosa, Poçem; 40.509639°N, 19.731194°E; alt. 40 m a.s.l.; 13 May 2024; leg. WG; CKLP.

**Comments**. Previously reported from BG. First record from AL.

**21. *Paradelphomyia czizekiana* Starý, 1971**

**Material examined. North Macedonia** • 3 ♂♂; Novo Selo, Bistra Mts., Mavrovo NP; 41.719438°N, 20.82889°E; alt. 990 m a.s.l.; 29 June 2017; leg. LPK, ET; CKLP.

**Comments**. Previously reported from BG and GR. First record from MK.

**22. *Paradelphomyia senilis* (Haliday, 1833)**

**Material examined. Albania** • 1 ♂; Tepelenë, Bënçë River; 40.287822°N, 20.008093°E; alt. 240 m a.s.l.; 10 October 2023; leg. LPK; CKLP.

**Comments**. Previously reported from BG, HR, GR, MK, TR. First record from AL.

**23. Rhabdomastix (Rhabdomastix) eugeni Starý, 2004**

**Material examined. Albania** • 2 ♂♂; Tepelenë, confluence of Vjosa and Drinos; 40.28034°N, 20.04429°E; alt. 130 m a.s.l.; 14 June 2014; leg. WG; CKLP.

**Comments**. Previously reported from BG and GR. First record from AL.

**24. Rhipidia (Rhipidia) ctenophora Loew, 1871**

**Material examined. Albania** • 1 ♂; Petran, Lëngarica Canyon; 40.2499°N, 20.4448°E; alt. 350 m a.s.l.; 12 October 2023; leg. LPK; CKLP.

**Comments**. Previously reported from BG and GR. First record from AL.

**25. *Rhypholophus bifurcatus* Goetghebuer, 1920**

**Material examined. Montenegro** • 2 ♂♂; Premćani, Premćanski Most (bridge); 43.1133°N, 19.3609°E; alt. 670 m a.s.l.; 5 October 2023; leg. LPK; CKLP.

**Comments**. Previously reported from BG, GR, XK, RS, and TR. First record from ME.

**26. Symplecta (Psiloconopa) alexanderi (Savchenko, 1973)**

Fig. 7

**Figure 7.**
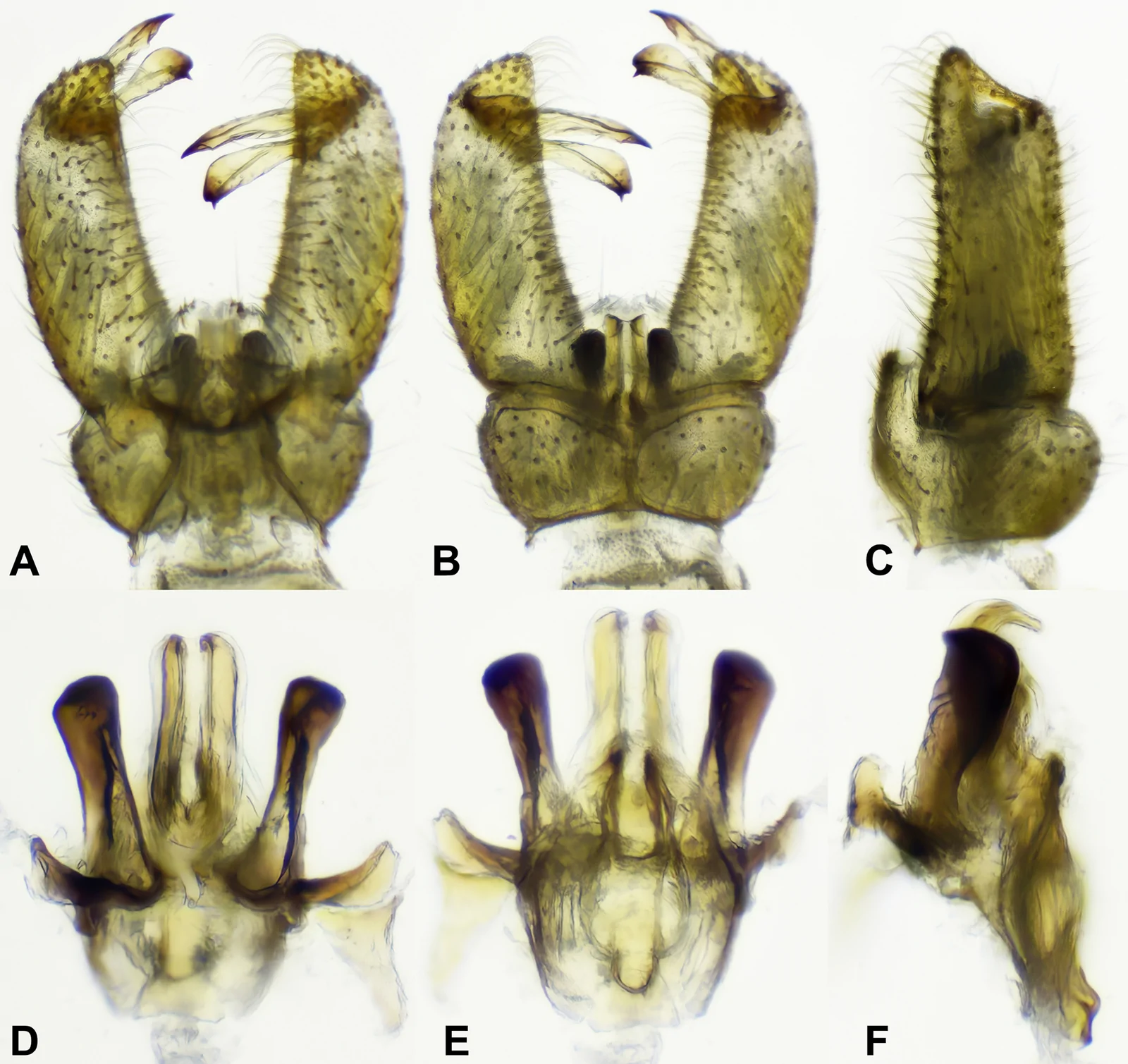
Symplecta (Psiloconopa) alexanderi (Savchenko, 1973) male: Albania, Poçem, CKLP: EULIM_11_064. **A–C**. Male terminalia. **A**. Dorsal view; **B**. Ventral view; **C**. Lateral view; **D–F**. Aedeagus complex; **D**. Dorsal view; **E**. Ventral view; **F**. Lateral view.

**Material examined. Albania** • 3 ♂♂; Tepelenë, confluence of Vjosa and Drinos; 40.28034°N, 20.04429°E; alt. 130 m a.s.l.; 14 June 2014; leg. WG; CKLP • 1 ♂; Poçem, Vjosa River; 40.50672°N, 19.72575°E; alt. 40 m a.s.l.; 14 June 2014; leg. WG; CKLP • 38 ♂♂; Vjosa, Poçem; 40.509639°N, 19.731194°E; alt. 40 m a.s.l.; 13 May 2024; leg. WG; CKLP • 1 ♂; Vjosa, Tepelenë Wald; 40.290861°N, 20.028139°E; alt. 125 m a.s.l.; 10 May 2024; leg. WG; CKLP.

**Comments**. Previously reported from MK. First records from AL.

**27. Symplecta (Symplecta) hybrida (Meigen, 1804)**

**Material examined. Albania** • 1 ♂; Vjosa; 40.292694°N, 20.024528°E; alt. 125 m a.s.l.; 27 March 2024; leg. AR; CKLP.

**Comments**. Previously reported from BiH, BG, HR, GR, ME, MK, RS, and the TR. First record from AL.

**28. Tasiocera (Dasymolophilus) exigua Savchenko, 1973**

**Material examined. Bosnia and Herzegovina** • 1 ♂; Republika Srpska, Sutjeska, forest spring outlet along the Tjentište–Ćurevo road; 43.35866°N, 18.70809°E; alt. 820 m a.s.l.; 4 May 2023; leg. LPK, TK, DM; CKLP.

**Comments**. Previously reported from RS. First record from BiH.

### Tipulidae

**29. Dolichopeza (Dolichopeza) nitida Mik, 1874**

**Material examined. North Macedonia** • 1 ♂, 1 ♀; Polog, Šar Planina, Popova Shapka, open stream above the village; 42.019194°N, 20.87975°E; alt. 1818 m a.s.l.; 25 June 2024; leg. TK, AA, KH; CKLP.

**Comments**. Previously reported from BG, GR, and RS. First record from MK.

**30. *Nephrotoma guestfalica guestfalica* (Westhoff, 1879)**

**Material examined. Albania** • 1 ♂; Vjosa; 40.640183°N, 19.401489°E; alt. 5 m a.s.l.; 23 April 2024; leg. WG; CKLP.

**Comments**. Previously reported from BiH, BG, HR, GR, MK, and TR. First record from AL.

**31. Tipula (Acutipula) ismene Mannheims, 1969**

**Material examined. Albania** • 1 ♂; Frashër, spring; 40.3442°N, 20.433956°E; alt. 1140 m a.s.l.; 11 October 2023; leg. LPK; CKLP. • 1 ♂, 1 ♀; Pagri, Kamenckës Canyon; 40.3278°N, 20.3655°E; alt. 950 m a.s.l.; 11 October 2023; leg. LPK; CKLP.

**Comments**. Only known from GR and MK. First records from AL.

**32. Tipula (Acutipula) tenuicornis Schummel, 1833**

**Material examined. Montenegro** • 3 ♂♂; Gusinje municipality, Gusinje, Alipaši Springs; 42.54918°N, 19.82473°E; alt. 940 m a.s.l.; 6 May 2023; leg. LPK, TK, DM; CKLP.

**Comments**. Previously reported from BiH, BG, HR, MK, and RS. First record from ME.

**33. Tipula (Pterelachisus) pabulina Meigen, 1818**

**Material examined. Montenegro** • 2 ♂♂; Plav municipality, Visitor Mts, Murino, Murinska Stream at the edge of the settlement; 42.6553°N, 19.88008°E; alt. 885 m a.s.l.; 5 May 2023; leg. LPK, TK, DM; CKLP.

**Comments**. Previously reported from BiH, BG, HR, MK, and RS. First record from ME.

**34. Tipula (Pterelachisus) pseudovariipennis Czizek, 1912**

**Material examined. Albania** • 1 ♂; Boge, Boge, Prokletje Mts.; 42.398206°N, 19.673917°E; alt. 1020 m a.s.l.; 2 May 2016; leg. LPK; CKLP. • 1 ♂; Sarande, Butrint, Butrint lake shore; 39.745429°N, 20.020297°E; alt. 12 m a.s.l.; 3 May 2016; leg. LPK; CKLP.

**Comments**. Previously reported from BiH, BG, HR, GR, ME, MK, RS, and TR. First record from AL.

**35. Tipula (Savtshenkia) aspromontensis Theowald, 1973**

**Material examined. Montenegro** • 1 ♂, 1 ♀; Rijeka Crnojevića, spring of Rijeka Crnojevića; 42.351725°N, 19.007986°E; alt. 45 m a.s.l.; 6 October 2023; leg. LPK; CKLP. **North Macedonia** • 1 ♂; Tresonche, Tresonce Waterfall; 41.5670°N, 20.7551°E; alt. 1340 m a.s.l.; 14 October 2023; leg. LPK; CKLP.

**Comments**. Previously reported from GR. First records from ME and MK.

**36. Tipula (Savtshenkia) benesignata Mannheims, 1954**

**Material examined. Albania** • 1 ♂; Frashër, Fir of Hotova National Park, springs; 40.344998°N, 20.395324°E; alt. 1100 m a.s.l.; 11 October 2023; leg. LPK; CKLP. • 3 ♂♂, 2 ♀♀; Pagri, Kamenckës Canyon; 40.3278°N, 20.3655°E; alt. 950 m a.s.l.; 11 October 2023; leg. LPK; CKLP.

**North Macedonia** • 2 ♂♂, 3 ♀♀; Tresonche, Tresonce Waterfall; 41.5670°N, 20.7551°E; alt. 1340 m a.s.l.; 14 October 2023; leg. LPK; CKLP.

**Comments**. Previously reported from BG, HR, GR, XK, and ME. First record from AL and MK.

**37. Tipula (Savtshenkia) cheethami Edwards, 1924**

**Material examined. Montenegro** • 1 ♂; Mojkovac municipality, Sinjajevina Mts, spring and outlet along road P4; 42.99265°N, 19.48441°E; alt. 910 m a.s.l.; 21 June 2023; leg. EKCU field practice; CKLP.

**Comments**. Previously reported from BiH, BG, HR, and GR. First record from ME.

**38. Tipula (Savtshenkia) gimmerthali gimmerthali Lackschewitz, 1925**

**Material examined. Albania** • 1 specimen; Frashër, spring; 40.3442°N, 20.433956°E; alt. 1140 m a.s.l.; 11 October 2023; leg. LPK; CKLP.

**Comments**. Previously reported from BG and MK. First record from AL.

**39. Tipula (Savtshenkia) grisescens Zetterstedt, 1851**

**Material examined. North Macedonia** • 1 ♂; Polog, Mavrovo and Rostuša, Bistra, Mavrovo, above the village; 41.64732°N, 20.74196°E; alt. 1280 m a.s.l.; 27 April 2021; leg. TK, DM, PO; CKLP.

**Comments**. Previously reported from AL, BiH, and ME. First record from MK.

**40. Tipula (Savtshenkia) obsoleta Meigen, 1818**

**Material examined. Montenegro** • 1 ♂; Gusinje, Grebaje Valley; 42.506687°N, 19.773344°E; alt. 1300 m a.s.l.; 4 October 2023; leg. LPK; CKLP.

**Comments**. Previously reported from BiH, HR, GR, and RS. First record from ME.

**41. Tipula (Savtshenkia) odontostyla Savchenko, 1961**

Fig. 8

**Figure 8.**
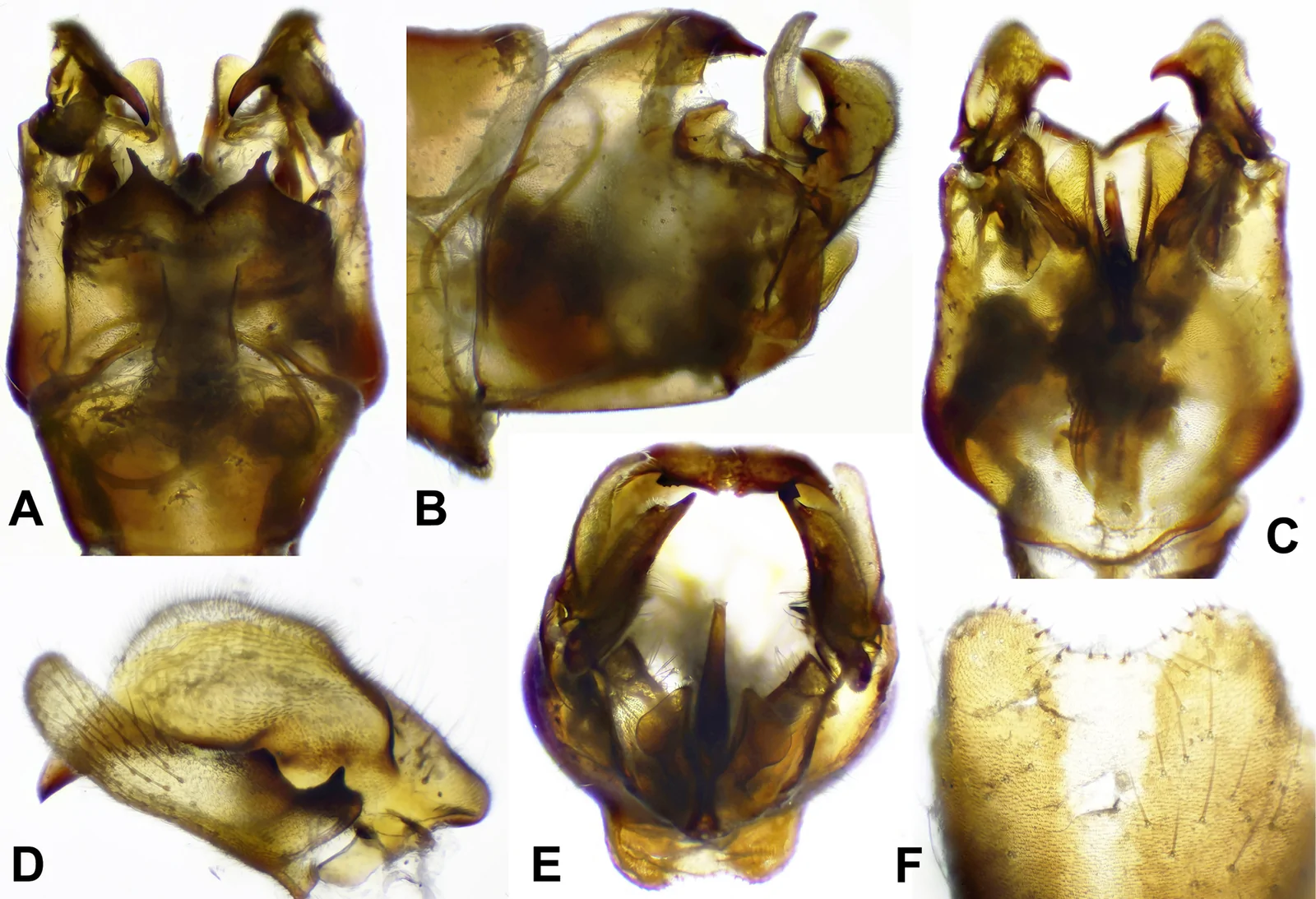
Tipula (Savtshenkia) odontostyla Savchenko, 1961 male: Bulgaria; Kalofer; CKLP: EUTIP_5_096. **A–C**, **E**. Male terminalia; **A**. Dorsal view; **B**. Lateral view; **C**. Postero-ventral view; **E**. Posterior view; **D**. Gonostylus, outer-lateral view; **F**. Sternite 8, ventral view.

**Material examined. Bulgaria** • 13 ♂♂, 3 ♀♀; Kalofer, Stara Planina Mts., Tundzha River; 42.669285°N, 24.987644°E; alt. 900 m a.s.l.; 28 October 2016; leg. LPK, ET; CKLP.

**Comments**. Previously reported from GR and TR. First record from BG.

**42. Tipula (Savtshenkia) pechlaneri Mannheims & Theowald, 1959**

Fig. 9

**Figure 9.**
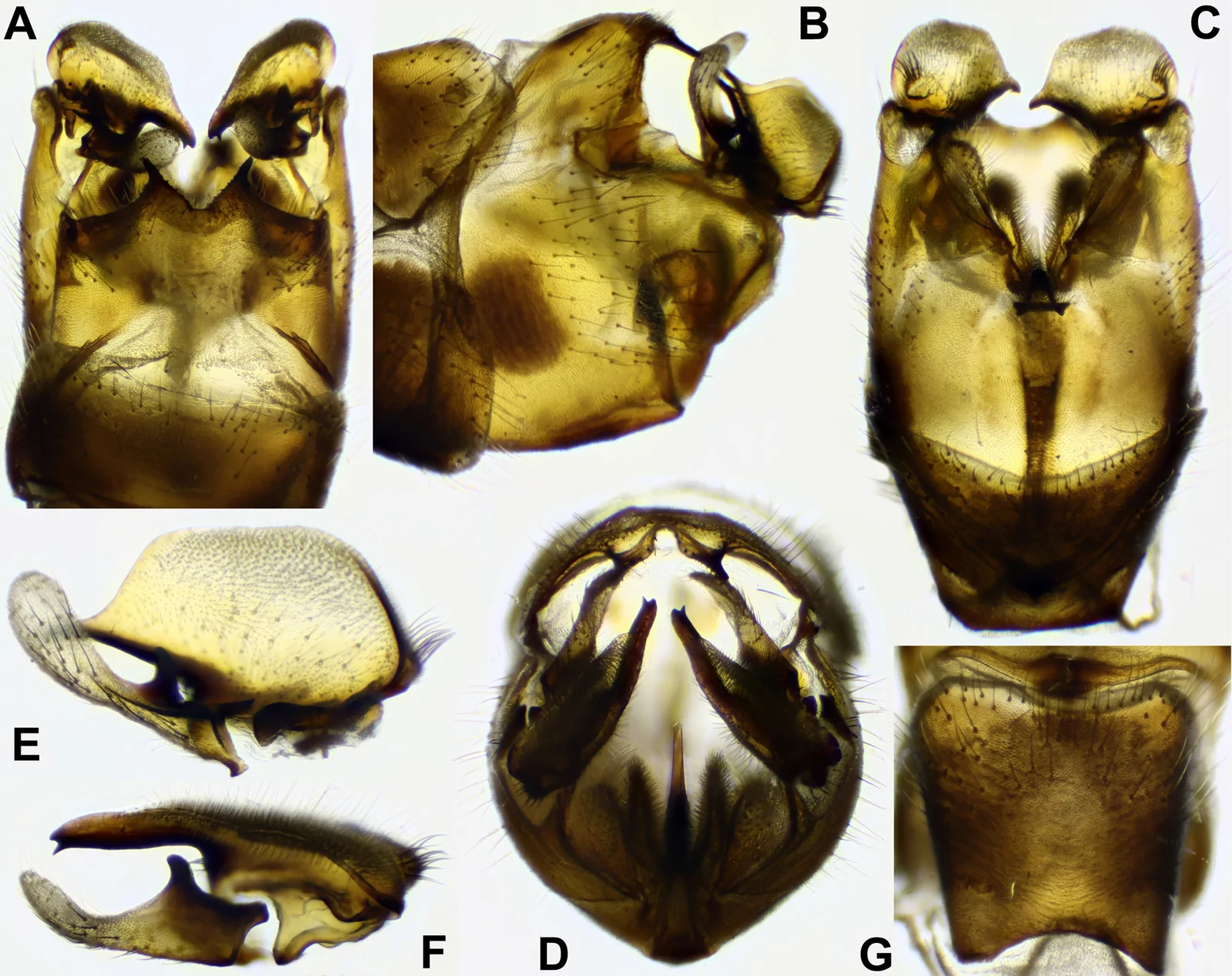
Tipula (Savtshenkia) pechlaneri Mannheims & Theowald, 1959 male: Serbia; Raška; CKLP: EUTIP_5_076. **A–D**. Male terminalia; **A**. Dorsal view; **B**. Lateral view; **C**. Postero-ventral view; **D**. Posterior view; **E**. Gonostylus, outer-lateral view; **F**. Gonostylus, posterior view; **G**. Sternite 8, ventral view.

**Material examined. Serbia** • 1 ♂; Raška, Novi Pazar, Golija Mts, Radaljica, spring brooks at forest edge; 43.2743°N, 20.3467°E; alt. 1575 m a.s.l.; 6 October 2025; leg. TK, AE, KF, DM; CKLP.

**Comments**. Previously reported from GR. First record from RS.

**43. Tipula (Savtshenkia) rufina rufina Meigen, 1818**

**Material examined. Montenegro** • 2 ♂♂, 1 ♀; Rijeka Crnojevića, spring of Rijeka Crnojevića; 42.351725°N, 19.007986°E; alt. 45 m a.s.l.; 6 October 2023; leg. LPK; CKLP.

**Comments**. Previously reported from BiH, BG, HR, GR, MK, and RS. First record from ME.

**44. Tipula (Tipula) italica errans Theowald, 1984**

**Material examined. Albania** • 1 ♂; Petran, Lëngarica Canyon; 40.2499°N, 20.4448°E; alt. 350 m a.s.l.; 12 October 2023; leg. LPK; CKLP.

**Montenegro** • 1 ♂; Rijeka Crnojevića, spring of Rijeka Crnojevića; 42.351725°N, 19.007986°E; alt. 45 m a.s.l.; 6 October 2023; leg. LPK; CKLP.

## Discussion

As previously noted by Kolcsár et al. (2023a, 2023b), the Balkan Peninsula remains the least investigated region of Europe in terms of crane fly faunistics. As a result, the discovery of species not previously recorded from one or any of the Balkan countries are relatively frequent.

Most of the species reported here are common and widespread in Europe, and their presence in the Balkans is best explained by the historical lack of faunistic research in the region rather than by recent dispersal events, which have been documented in some other European crane flies (Kolcsár 2025).

## Supplementary Material

XML Treatment for Dactylolabis (Coenolabis)

XML Treatment for Dactylolabis (Coenolabis) posthabita

XML Treatment for Dactylolabis (Coenolabis) posthabita posthabita

XML Treatment for Dactylolabis (Coenolabis) posthabita tothi

## Additional information

### Artificial Intelligence (AI) use

The authors accept full responsibility for the content of the manuscript, including the disclosure of any use of AI.

Regarding the use of AI in the preparation of this manuscript, the authors declare the following: GPT used for language, style and writing.

### Author ORCIDs

L.-P. Kolcsár https://orcid.org/0000-0001-7784-2386

W. Graf https://orcid.org/0000-0001-6559-0644

T. Kovács https://orcid.org/0000-0003-4577-0394

D. Murányi https://orcid.org/0000-0002-3907-5590

M. Ivković https://orcid.org/0000-0003-3188-5676

### Data availability

All of the data that support the findings of this study are available in the main text.
